# Sleepiness and cognition in young adults who gamble and use alcohol

**DOI:** 10.1556/JBA.3.2014.014

**Published:** 2014-08-26

**Authors:** ARIT M. HARVANKO, KATHERINE L. DERBYSHIRE, R.N. LIANA SCHREIBER, JON E. GRANT

**Affiliations:** ^1^Department of Psychology, University of Kentucky College of Arts and Sciences, Lexington, KY, USA; ^2^Department of Psychiatry & Behavioral Neuroscience, University of Chicago, Pritzker School of Medicine, Chicago, IL, USA; ^3^Division of Epidemiology and Community Health, University of Minnesota, Minneapolis, MN, USA

**Keywords:** sleep, gambling disorder, cognition, decision-making, alcohol

## Abstract

*Background and aims:* Past research suggests that sleep problems are associated with increased risky decision-making. Similarly, gambling disorder and alcohol use disorder are also associated with increased risky decision-making. Individuals with gambling disorder or alcohol use disorder have also reported higher rates of sleep problems compared to normal healthy controls. As such, we sought to examine whether sleep problems play a role in the development of alcohol use disorder or gambling disorder. *Methods:* One hundred and forty-one individuals who gamble and use alcohol, yet do not meet criteria for gambling disorder or alcohol use disorder, were assessed to determine the correlation between sleepiness, amount of sleep obtained, decision-making, and alcohol or gambling behaviors. *Results:* Our results suggest that inconsistent sleep patterns may be associated with increased frequency of alcohol use and gambling. We did not, however, find a significant correlation between sleep factors and decision-making. *Discussion:* Further research is needed to examine the specific relationship between sleep patterns and alcohol use and gambling frequency. Overall these data suggest that sleepiness or sleep and risky decision-making is not a significant factor in gambling and alcohol use behaviors in individuals not meeting criteria for alcohol use disorder or gambling disorder.

## Introduction

It has been estimated that between 0.6-4.8% of individuals in the United States have met criteria for gambling disorder (GD)at some point in their lives (Kessler et al., 2008; Welte, Barnes, Wieczorek, Tidwell & Parker, 2001). It has been estimated that 30.3% of individuals in the United States have met criteria for alcohol use disorder (AUD)at some point in their lives (Hasin, Stinson, Ogburn & Grant, 2007). There is consensus amongst the literature that alcohol and gambling problems are somehow correlated. For example, Welte et al. (2001)found that the odds ratio of an individual meeting criteria for current alcohol use disorder and current gambling pathology was 23.1 in a large community sample, while other research has found a similarly significant relationship between the prevalence of alcohol and gambling problems (Barnes, Welte, Hoffman & Tilde, 2009; Crockford & el-Guebaly, 1998; Smart & Ferris, 1996). In addition to having high comorbidity, it has been suggested that alcohol and gambling problems may have similar causes or consequences. Specifically, individuals with AUD and GD have demonstrated similar cognitive deficits (compared to normal controls)(Cavedini, Riboldi, Keller, D’Annucci & Bellodi, 2002; Goudriaan, Oosterlaan, de Beurs & van den Brink, 2006; Lawrence, Luty, Bogdan, Sahakian & Clark, 2009; van Holst, van den Brink, Veltman & Goudriaan, 2010), which may suggest a common cognitive predisposition towards AUD or GD behaviors, or a similar cognitive deficit resultant of AUD or GD associated behaviors.

One factor involved in AUD or GD associated cognitive deficits may be sleep. With alcohol, trouble sleeping in childhood has been found to predict early onset of alcohol use (Wong, Brower & Zucker, 2009). Also, persons with an alcohol use disorder, who experience greater sleep problems after abstaining from alcohol, have shown significantly high rates of relapse (Brower, Aldrich & Hall, 1998; Gillin et al., 1994). Although previous research has examined health issues in individuals with gambling disorder (Desai, Maciejewski, Dausey, Caldarone & Potenza, 2004; Erickson, Molina, Ladd, Pietrzak & Petry, 2005; Pasternak and Fleming, 1999; Scherrer et al., 2005), few studies have examined sleep problems and gambling disorder.

Lack of sleep itself may produce or exacerbate some of the cognitive problems associated with AUD and GD. Research has shown that healthy individuals, when deprived of sleep, begin to show decision-making problems similar to those seen in individuals with gambling disorder. Killgore, Balkin & Wesensten (2006)found that after 49.5 hours of continual wakefulness healthy adults began to make increasingly risky and disadvantageous choices on the Iowa Gambling Task. Research has also shown that after 24 hours of continual wakefulness neural processing patterns while performing a gambling task significantly change in a manner that may suggest a propensity towards increased risk seeking (Venkatraman, Chuah, Huettel & Chee, 2007). In addition, fatigue (measured using the Piper Fatigue Scale)appears to have a significant positive correlation with the amount of risk taken in a decision-making task (defined as amount of money bet during a gambling task)(Frings, 2012). Considered together, these studies suggest that prolonged and continual wakefulness, fatigue, or accumulated sleep debt may increase risky decision-making in individuals who gamble.

Although the topic of sleep has largely been absent from gambling research, two studies examining sleep and GD have recently been published. Parhami et al. (2012)examined the relationship between sleep (using the Pittsburgh Sleep Quality Index, the Epworth Sleepiness Scale [ESS]), and gambling severity (based on number of GD diagnostic criteria met). Sleepiness, assessed with the ESS, showed a significant positive correlation with gambling pathology (i.e. from recreational to gambling disordered individuals). As the authors mention, this study did not account for psychiatric comorbidity, which is quite common in GD (Petry, Stinson & Grant, 2005). Addressing this issue, Parhami, Siani, Rosenthal & Fong (2013)used data collected from a national survey to investigate the correlation between sleep and gambling problems. Approximately 45.9% of the individuals who reported problematic gambling behavior reported at least one sleep problem. After controlling for age and psychiatric comorbidity, problem gamblers were significantly more likely to report difficulty initiating sleep compared to recreational gamblers, while individuals with GD reported difficulty initiating and maintaining sleep, and awaking earlier than desired in the morning. These two studies suggest that sleep disturbance of some type is likely associated with gambling behaviors.

The aforementioned literature suggests that gambling behaviors are somehow interrelated with sleep problems, which in turn are associated with risky decision-making. These relationships, however, have yet to be examined in a single study. Based on previous evidence, we hypothesize that sleep problems play a key role in early stages of the development of GD through its influence on decision-making. Therefore, we believe that there will be significant correlations between sleep variables, risky decision-making, and GD criteria met in individuals who gamble but do not qualify for a diagnosis of GD. Based on the comorbidity and common psychopathology of AUD and GD, we also believe that this correlation will be similar to the correlation between alcohol use, sleep, and decision-making. To test these hypotheses, the current study was designed to collect data on sleepiness (as defined by Johns, 1991), amount of sleep received (on average and the night prior to testing), gambling behavior (including GD criteria met), and alcohol use behavior, with an analysis constructed to determine whether sleep, gambling and alcohol use behaviors, and risky decision-making are synergistically related.

## Methods

### Participants

One hundred and forty-one young adults (18-29 years of age)from two Midwestern metropolitan areas voluntarily participated in a study on impulsivity. Participants were recruited using media advertisements and referrals from previous study participants. Study participants completed their visits within offices on the campuses of the Universities of Minnesota and Chicago. Participants were compensated with $50 gift cards to a nationwide department store. Additional compensation of two bus tokens (if public transportation was used to arrive at the study location)or parking vouchers was also offered. Study visits lasted approximately two hours and participants were allowed to take breaks as necessary.

Requirements for entering the study included having gambled five times in the past 12 months, being within ages 18-29 years, being able to understand and sign the study consent form, and being able to perform all study procedures. Individuals who endorsed more than three GD criteria or met criteria for AUD were excluded from this study.

### Measures

The Epworth Sleepiness Scale (ESS) (Johns, 1991), a valid and reliable scale of sleepiness, was used as a measure of sleepiness. ESS scores have been shown to positively correlate with GD criteria (Parhami et al., 2012)and negatively correlate with executive functioning (Anderson, StorferIsser, Taylor, Rosen & Redline, 2009; Naismith, Winter, Gotsopoulos, Hickie & Cistulli, 2004). The ESS poses eight hypothetical situations, which the test taker rates in terms of how likely they would be to fall asleep in them: (0) no-, (1)slight-, (2)moderate-, (3)high-chance of dozing. Total scores, ranging from 0-24, are used for evaluation of sleepiness in this study.

The Mini International Neuropsychiatric Inventory (MINI)(Sheehan et al., 1998)is a clinical screening tool used to assess presence of criteria for psychiatric disorders.

The Structured Clinical Interview for Pathological Gambling (SCI-PG)(Grant, Steinberg, Kim, Rounsaville & Potenza, 2004) is a valid and reliable interviewing tool based on diagnostic criteria for GD. This is a 10-item questionnaire, with each item representing a criterion for GD.

The Alcohol Use Disorders Identification Test (AUDIT)(Saunders, Aasland, Babor, de la Fuente & Grant, 1993) is a well-validated 10-item questionnaire used to assess alcohol use behaviors and potential problems. Each item is scored 0-4, with a total of 0-40 points possible. Saunders et al. (1993)suggest that a score of higher than eight likely indicates hazardous or harmful alcohol use.

### Decision-making assessment

In addition to completing a guided interview, participants were asked to complete the Cambridge Gamble Task (CGT)from the Cambridge Neuropsychological Automated Test Battery (CANTABeclipse, version 3, Cambridge Cognition Ltd, UK). The CGT is a valid and reliable computerized cognitive task used to assess the active decision-making process with a gambling paradigm. Clark et al. (2004)have previously described the CGT in detail.

Three variables from the CGT were used in this study. (1) Overall proportion of points gambled (a measure of risk taking), which has been shown to positively correlate with prefrontal cortical damage (Clark, Cools & Robbins, 2004), at-risk alcohol use and alcohol use disorder (Harvanko, Odlaug, Schreiber & Grant, 2012), and GD (Lawrence et al., 2009). (2) Quality of decision-making (calculated as the proportion of probabilistically likely decisions chosen), which has been shown to negatively correlate with damage in the orbitofrontal areas of the brain. (3) Deliberation time (time it took to determine which color to select), which has been shown to positively correlate with diagnosis of alcohol use disorder (Lawrence et al., 2009).

### Procedure

Participants completed study visits in research clinics at the Universities of Minnesota and Chicago. Data presented in this analysis were collected as part of an ongoing longitudinal study examining impulsivity in young adults. As part of this study, participants completed in-office visits at baseline, and annually (for up to 5 years). In addition to the measures presented in the current analysis, participants completed a variety of other self-report and interviewer guided personality measures and computerized cognitive measures. On average, study visits took approximately two and a half hours to complete. The ESS was not introduced into the study initially, therefore the first completion of the ESS may have occurred at baseline or a later annual visit for some participants. Only data from the first visit participants completed the ESS on was used from each participant.

### Statistical analysis

Statistical comparisons were made using regression analysis and Pearson’s correlation coefficient tests. All data were analyzed using SPSS version 21.0 (SPSS IBM, New York, USA). Based on previous research suggesting that marijuana use is significantly related to sleep and alcohol and gambling behavior (Schierenbeck, Riemann, Berger & Hornyak, 2008), the participants’ self-reports of marijuana use was used as a covariate in the analysis. Participants did not report use of any illicit drugs besides marijuana. The intrinsic variables age and gender were included as potential sources of variance in each regression model. Alcohol use frequency and AUDIT scores were controlled for when examining gambling variables, while gambling frequency and SCI-PG scores were controlled for when analyzing alcohol variables. Lifetime prevalence of axis-I psychiatric disorders and lifetime prevalence of an impulse control disorder were included as known correlates of alcohol and gambling behavior.

### Ethics

The respective institutional review boards at the Universities of Minnesota and Chicago approved all study procedures. The ethical principles outlined in the Declaration of Helsinki were followed throughout the conduct of this study. All study participants provided informed consent before participating in study procedures.

## Results

A total of 141 participants were found to have complete data and included in the analysis. Overall sample demographics, gambling, alcohol, and drug use variables, and psychiatric history can be found in Table 1.

Overall reported lifetime prevalence of axis-I disorders was: major depressive disorder (*n* = 32), substance abuse (*n* = 11), bipolar disorder (*n* = 9), antisocial personality disorder (*n* = 7), generalized anxiety disorder (*n* = 2), panic disorder (*n* =1), post-traumatic stress disorder (*n* = 1), and obsessive-compulsive disorder (*n* = 1) with some participants endorsing more than one axis-I disorder. Overall reported lifetime prevalence of impulse control disorders was: compulsive sexual behavior (*n* = 4), compulsive buying (*n* = 4), eating disorders (*n* = 2), intermittent explosive disorder (*n* = 2), and trichotillomania (*n* = 1).

**Table 1.** Demographic and clinical variables

	Total sample *N* = 141
Age (years), mean ± *SD*	23.38 ±3.32
Gender
Female, *N* (%)	61 (43.26)
Education
High school or less, *N* (%)	5 (3.55)
Some college, *N* (%)	79 (56.03)
College graduate or more, *N* (%)	57 (40.43)
Relationship Status
Single, *N* (%)	134(95.04)
Married, *N* (%)	7 (4.96)
Race/Ethnicity
Caucasian, *N* (%)	113 (80.14)
Other, *N* (%)	28 (19.86)
Sexual Orientation
Heterosexual, *N* (%)	129 (91.49)
Other, *N* (%)	12(8.51)
Gambling Frequency
Sessions per week, mean ± *SD*	1.11 ± 1.82
Alcohol Use
Drinks per week, mean ± *SD*	1.51 ± 1.14
Alcohol Use Disorders Identification Test
Total score, mean ± *SD*	6.55 ±4.80
Structured Clinical Interview for Pathological Gambling
Number of criterion present, mean ± *SD*	.25 ± .55
Marijuana Use
Times per week, mean ± *SD*	0.47 ± 1.36
Lifetime Axis-I Disorder(s), *N* (%)	37 (26.06)
Lifetime Impulse Control Disorder(s), *N* (%)	45 (31.69)
Epworth Sleep Scale Total, mean ± *SD*	7.04 ±4.04
Hours of Sleep Last Night, mean ± *SD*	6.72 ± 1.62
Average Hours of Sleep, mean ± *SD*	7.08 ± 1.14

Bivariate correlational analysis was done on all study variables to be used in subsequent analysis (Table 2). Regression models were used for the primary analysis. To test for possible multicollinearity issues variance inflation scores were calculated for the set of independent variables used in each model, with a variance inflation score threshold of 10 or greater indicating a significant multicollinearity issue. Variance inflation scores ranged from 1.01-1.45, and were therefore not indicative of a significant multicollinearity issue. Each model used a unique dependent variable (i.e., gambling frequency, SCI-PG score, alcohol use frequency, and AUDIT score) with the same independent variables used throughout. Results of this analysis are presented in Table 3.

Analysis of interactions between sleep variables and gambling (i.e. gambling frequency and SCI-PG score)and alcohol (i.e. alcohol use frequency and AUDIT score)variables was performed using the cross product of the six cognitive and sleep related variables (i.e. ESS total, hours of sleep last night, hours of sleep on average, CGT deliberation time, CGT quality of decision-making, and CGT Overall proportion bet)analyzed with Pearson’s correlation coefficient analysis. After family-wise error correction using Hochberg’s step-up procedure (Benjamini & Hochberg, 1995), none of these correlations were significant.

**Table 2.** Pearson correlation matrix of study variables

		1	2	3	4	5	6	7	8	9	10	11	12	13	14	15
1.	Age															
2.	Gender	.559														
3.	Lifetime Axis-I Disorder	.244**	-.008													
4.	Lifetime Impulse Control Disorder	.175*	-.026	.422**												
5.	Frequency of Marijuana Use	.116	-.018	.380**	.305**											
6.	Gambling Frequency	.246**	.048	.251**	.389**	.304**										
7.	SCI-PG Score	.142	-.111	.269**	.269**	.318**	.337**									
8.	Alcohol Use Frequency	.009	.098	.049	.048	.283**	-.106	-.118								
9.	AUDIT Score	.126	.350**	.033	-.005	.085	.027	.014	.360**							
10.	Epworth Sleepiness Scale Total	-.209*	-.081	.063	.009	.039	-.003	.128	-.042	-.029						
11.	Hours of Sleep Last Night	-.104	-.005	-.035	-.139	-.089	.083	.005	.111	.032	-.033					
12.	Average Hours of Sleep	.003	.005	.035	-.029	.055	.064	.089	.027	.008	-.145	.371**				
13.	CGT Deliberation Time	.224**	-.074	.147	.230**	.189*	.156	-.035	.068	-.036	-.003	-.162	-.034			
14.	CGT Quality of Decision Making	-.104	.152	.028	-.128	-.134	-.070	-.194*	.064	.052	.022	-.118	-.163	-.309**		
15.	CGT Overall Proportion Bet	.151	.178*	.103	.216*	.092	.115	.036	-.021	.142	.071	-.069	.032	.170*	-.004	

CGT = Cambridge Gamble Task; SCI-PG = Structured Clinical Interview for Pathological Gambling; AUDIT = Alcohol Use Disorders Identification Test; * Significance with *p* ≤ .05; ** Significance with *p* ≤ .01

**Table 3.** Summary of regression on gambling behavior and alcohol use measures

Predictor				Model I (DV = Gambling Frequency)					Model II (DV = SCI-PG Score)								Model III (DV = Alcohol Use Frequency)							Model IV (DV = AUDIT Score)				
		*B*		*SE B*			*ß*		*B*		*SE B*			*ß*		*B*	*SE B*		*ß*			*B*		*SE B*			*ß*	
Age		**.103**		**.045**			.189*		.013		.010			.104		.025	.030		.072			.240		.132			.166	
Gender		.307		.299			.084		-.064		.068			.079		.246	.189		.107			**3.375**		**.822**			.349***	
Lifetime Axis-I Disorder		-.140		.365			-.034		.091		.083			.100		-.199	.244		-.077			-.306		1.063			-.028	
Lifetime Impulse Control Disorder		**1.272**		**.335**			.328**		.142		.076			.166		.295	.235		.121			-.246		1.022			-.024	
Frequency of Marijuana Use		**.377**		**.116**			.283**		**.082**		**.026**			.278**		**.350**	**.076**		420***			.390		.330			.111	
Gambling Frequency																-.156	.059		-.249**			-.162		.255			-.061	
SCI-PG Score																-.412	.263		-.145			.328		1.142			.027	
Alcohol Use Frequency		**-.416**		**.136**			-.261**		**-.074**		**.031**			.209*														
AUDIT Score		.016		.033			.043		.007		.007			.086														
Epworth Sleepiness Scale Total		.014		.035			.030		.013		.008			.133		-.002	.024		-.006			.025		.103			.021	
Hours of Sleep Last Night		**.247**		**.094**			.220**		.004		.021			.017		**.157**	**.063**		.223*			.211		.273			.071	
Average Hours of Sleep		.008		.130			.005		.022		.029			.062		-.019	.087		-.019			-.092		.377			-.022	
Cambridge Gamble Task																												
Deliberation Time		<.001		<.001			.082		**<.001**		**<.001**			.190*		<.001	<.001		.087			<.001		<.001			-.043	
Quality of Decision Making		2.163		2.147			.084		-.890		.486			.156		2.164	1.448		.133			1.426		6.296			.021	
Overall Proportion Bet		-4.53		1.071			-.034		-.098		.243			.033		-.602	.709		-.071			2.480		3.084			.070	
Adjusted *R^2^*				.235							.193						.136							.079				

DV = dependent variable; SCI-PG = Structured Clinical Interview for Pathological Gambling; AUDIT = Alcohol Use Disorders Identification Test; * Significance with *p* ≤ .05; ** Significance with *p* ≤ .01; *** Significance with *p* ≤ .001

## Discussion

In this study we examined sleeping habits, sleepiness, and cognitive decision-making performance in a sample ofindividuals who gamble and use alcohol. We found a significant relationship between hours of sleep last night, and gambling and alcohol use frequency. We also found a significant relationship between the CGT variables ‘decision-making’ and ‘deliberation time’, and SCI-PG score. We didnot, however, find that sleep problems and decision-making deficits had a significant interaction that correlated positively with gambling or alcohol related variables. Thus, our hypothesis that deficits in sleep or decision-making would have a synergistic effect that positively correlated with gambling or alcohol use frequency and behaviors was largely unsupported.

We did find that hours of sleep received the night prior to testing correlated with gambling and alcohol use frequency. Yet, the direction of this relationship was contrary to our expectations, with gambling or alcohol use frequency sharing a positive correlation with amount of sleep received the night prior to testing. It should be noted that this variable was primarily included in the analysis to ensure that sleepiness (ESS total score)or poorer cognitive performance was not simply indicative of one atypical night of less sleep, but rather average sleep duration or sleepiness. Nonetheless, this finding may suggest that individuals who use alcohol or gamble with a greater frequency than their peers may have greater fluctuations in sleep durations. These fluctuations could be resultant of decreased sleep after prolonged nights of gambling or alcohol use on previous days, which are compensated for by longer nights of sleep on other days. We could speculate that, since data collection sessions occurred during weekdays, individuals may have used alcohol or gambled on weekend nights, resulting in longer compensatory sleep duration on weekdays prior to data collection. Unfortunately, since individuals were only asked to self-report their average hours of sleep, we were not able to investigate this question. Therefore, future studies may want to examine whether there is a correlation between greater sleep duration variance over multiple nights and gambling or alcohol use.

Our finding that ESS total scores did not significantly correlate with SCI-PG scores is consistent with previous research on sleep and gambling severity. For example, Parhami et al. (2012)examined three groups of gamblers: recreational, problem, and pathological gamblers, having met 0, 1-4, and 5 or more GD criteria, respectively. They found that pathological gamblers had significantly higher ESS scores than problem and recreational gamblers. They did not, however, find that problem gamblers had significantly greater ESS scores than recreational gamblers. Since the data we present includes only individuals who could be considered ‘recreational’ or ‘problem’ gamblers, the lack of significant correlation between ESS total scores and SCI-PG criteria replicates this finding.

We did not find significant correlations between the CGT variables we examined and average hours of sleep per night, hours of sleep the night prior to testing, or sleepiness (as represented by the ESS). This finding may contrast previous research examining a similar question. Frings (2012)examined the effect of experimentally attenuated sleep duration (< 5 hours), over the course of two nights, on decision-making. Frings found that individuals who had experimentally reduced sleep duration bet significantly more on a probabilistic decision-making task compared to those whose sleep patterns were not manipulated. In the data we present, however, there were no significant correlations between sleep variables and amount bet on a probabilistic decision-making task (the CGT). This may be resultant of the difference between experimentally reduced sleep patterns (as in Frings, 2012) and the natural sleep differences we observed in this study, which could simply be reflective of random individual differences in sleep duration. This may suggest that, although maladaptive decision-making behavior may result from acute induced sleep deficits, these deficits do not appear to naturally occur with a high degree of prevalence in the gamblers of the severity sampled in this study. It should be noted, however, that the data we present does not address cognitive deficits that may occur during prolonged gambling sessions or gambling sessions occurring late at night. As such, individuals who gamble after extended periods of wakefulness may still be prone to make disadvantageous decisions despite the data we present.

While primarily interested in examining sleep problems in gamblers, we also analyzed alcohol use behaviors in relation to sleep as a reference point. The similarity in findings between alcohol and gambling frequency was unsurprising considering that alcohol use and gambling frequency are highly correlated in other research (Barnes et al., 2009; Crockford & el-Guebaly, 1998; Smart & Ferris, 1996)as well as in this sample. While statistically controlling for this correlation, however, we also found similar significant explanations of variance for gambling and alcohol use frequency (i.e. hours of sleep last night, frequency of marijuana use). Yet, we were surprised to find that sleep or cognitive variables were (with the exception of sleep last night and gambling frequency)not correlated with alcohol use variables. This may be due to the relatively less problematic alcohol use occurring in this sample (i.e. not having met criteria for AUD), or because the individuals in this sample vary in some way (e.g. environmental or social variables)that was unaccounted for in this study.

### Limitations

The authors would like to acknowledge a few limitations of this study. Many variables used in the analysis were derived from self-report. As such, frequency or severity of drug use, alcohol use, or gambling frequency may be under- or over-reported. Although we did not intentionally seek out college students, a disproportionately large number of individuals in this sample are, or were, a college student (as shown in Table 1).

## Conclusions

A sample of young adults who gamble and use alcohol were evaluated for sleep and cognitive problems associated with gambling and alcohol use behaviors. Sleep issues or cognitive deficits were largely unrelated to gambling and alcohol use frequency or problems. Yet, amount of sleep received the night prior to testing did have a significant correlation with alcohol use and gambling frequency. Overall, this study suggests that there is not a significant interaction between sleep, sleepiness, and risky decision-making in young adults who gamble and use alcohol. Since there was a significant effect of hours of sleep received prior to the night of testing, future research may want to examine sleep patterns over multiple days or weeks to investigate whether gambling or alcohol use frequency is associated with inconsistent nightly sleep durations.
